# Use of Eltrombopag to Improve Thrombocytopenia and Tranfusion Requirement in Anti-CD19 CAR-T Cell-Treated Patients

**DOI:** 10.3390/jcm13175117

**Published:** 2024-08-28

**Authors:** Maria-Eva Mingot-Castellano, Juan Luis Reguera-Ortega, Denis Zafra Torres, Rafael Hernani, Oriana Lopez-Godino, Manuel Guerreiro, Blanca Herrero, Lucia López-Corral, Alejandro Luna, Lesli Gonzalez-Pinedo, Anabelle Chinea-Rodriguez, Ana Africa-Martín, Rebeca Bailen, Nuria Martinez-Cibrian, Pascual Balsalobre, Silvia Filaferro, Anna Alonso-Saladrigues, Pere Barba, Antonio Perez-Martinez, María Calbacho, Jose Antonio Perez-Simón, Jose Maria Sánchez-Pina, on behalf of the Spanish Group of Hematopoietic Transplant and Cell Therapy (GETH-TC)

**Affiliations:** 1 Hospital Universitario Virgen del Rocío, Instituto de Biomedicina de Sevilla, IBiS/CSIC, Universidad de Sevilla, 41004 Sevilla, Spain; juanlu_jlr@hotmail.com (J.L.R.-O.); josea.perez.simon.sspa@juntadeandalucia.es (J.A.P.-S.); 2 Hospital Universitario 12 de Octubre, 28041 Madrid, Spainjosemspina@gmail.com (J.M.S.-P.); 3 INCLIVA Health Research Institute, Hospital Clínico Universitario, 46010 Valencia, Spain; 4 Hematology Department, Centro Regional de Hemodonación, IMIB-Pascual Parrilla, Hospital Universitario Morales-Meseguer, 30008 Murcia, Spain; orilopezgodino@gmail.com; 5 Servicio de Hematología, Hospital Universitario y Politécnico La Fe, 46026 Valencia, Spain; 6 Hospital Infantil Universitario del Niño Jesús, 28009 Madrid, Spain; blanca.herrero@salud.madrid.com; 7 IBSAL, CIBERONC, Centro de Investigación del Cáncer-IBMCC (USAL-CSIC), Hospital Universitario de Salamanca (Spain), 37007 Salamanca, Spain; lucialopezcorral@usal.es (L.L.-C.); aamartin@saludcastillayleon.es (A.A.-M.); 8 Hospital Universitario Ramón y Cajal, 28034 Madrid, Spain; 9 Hospital Universitario de Gran Canaria Dr. Negrín, 35010 Las Palmas de Gran Canaria, Spain; 10 Instituto de Investigación Sanitaria Gregorio Marañón, Hospital General Universitario Gregorio Marañón, 28009 Madrid, Spain; 11 Hospital Clinic Barcelona, Institut de Recerca Sant Joan de Déu, Barcelona Hospital Sant Joan de Déu, 08950 Barcelona, Spain; nmartinc@clinic.cat; 12 University Hospital Vall d’Hebron, 08035 Barcelona, Spain; pbalsalobre@geth.es (P.B.); sfilaferro@geth.es (S.F.); 13 Institut de Recerca Sant Joan de Déu, Hospital Sant Joan de Déu, 08950 Barcelona, Spain; anna.alonso@sjd.es; 14 Grupo Español de Trasplante Hematopoyético y Terapia Celular, 28040 Madrid, Spain; pbarba@vhio.net; 15 Pediatric Hematology-Oncology Department, La Paz University Hospital, 28034 Madrid, Spain; aperezmartinez@salud.madrid.org; 16 Pediatric Department, Autonomous University of Madrid, 28034 Madrid, Spain; 17 CIBERER-ISCIII, IdiPAZ-CNIO Pediatric OncoHematology Clinical Research Unit, 28034 Madrid, Spain

**Keywords:** CAR-T, thrombocytopenia, platelet transfusion, neutropenia, red blood cell transfusion, pan-cytopenia, thrombopoietin receptor agonist, eltrombopag, adverse events

## Abstract

**Background/Objectives:** Immune effector cell-associated hematotoxicity (ICAHT) is a frequent adverse event after chimeric antigen receptor (CAR)-T cell therapy. Grade ≥ 3 thrombocytopenia occurs in around one-third of patients, and many of them become platelet transfusion-dependent. Eltrombopag is a thrombopoietin receptor agonist (TPO-RA) able to accelerate megakaryopoiesis, which has been used successfully in patients with bone marrow failure and immune thrombocytopenia (ITP). Its role in managing thrombocytopenia and other cytopenias in CAR-T cell-treated patients has been scarcely addressed. Our aim was to report the safety and efficacy of this approach in patients included in the Spanish Group for Hematopoietic Transplantation and Cellular Therapy (GETH-TC) registry. **Methods:** This is a retrospective, multicenter, observational study. Patients who developed platelet transfusion dependence subsequently to CAR-T cells and received eltrombopag to improve platelet counts were recruited in 10 Spanish hospitals. **Results:** Thirty-eight patients were enrolled and followed up for a median (interquartile range [IQR]) of 175 (99, 489) days since CAR-T cell infusion. At the moment eltrombopag was indicated, 18 patients had thrombocytopenia and another severe cytopenia, while 8 patients had severe pancytopenia. After 32 (14, 38) days on eltrombopag, 29 (76.3%) patients recovered platelet transfusion independence. The number of platelet units transfused correlated with the time needed to restore platelet counts higher than 20 × 10^9^/L (Rho = 0.639, *p* < 0.001). Non-responders to eltrombopag required more platelet units (58 [29, 69] vs. 12 [6, 26] in responders, *p* = 0.002). Nineteen out of twenty-three (82.6%) patients recovered from severe neutropenia after 22 (11, 31) days on eltrombopag. Twenty-nine out of thirty-five (82.9%) patients recovered red blood cell (RBC) transfusion independence after 29 (17, 44) days. Seven patients recovered all cell lineages while on treatment. No thromboembolic events were reported. Only two transient toxicities (cholestasis, hyperbilirubinemia) were reported during eltrombopag treatment, none of which compelled permanent drug withdrawal. **Conclusions:** Eltrombopag could be safely used to manage thrombocytopenia and accelerate transfusion independence in CAR-T cell-treated patients.

## 1. Introduction

Therapies with chimeric antigen receptor (CAR)-T cells have dramatically changed treatment for patients with relapsed/refractory non-Hodgkin lymphoma (NHL), acute lymphoblastic leukemia (ALL), and multiple myeloma (MM) [1,2,3]. Furthermore, its use in patients with other malignancies and autoimmune diseases beyond current indications is a field of active research [4,5]. Cytokine release syndrome (CRS) and immune effector cell-associated neurotoxicity (ICANS) have been acknowledged as potentially life-threatening complications after CAR-T cell therapy, and guidelines for their management are available [6]. However, therapeutic measures to treat immune effector cell-associated hematotoxicity (ICAHT) have not been fully agreed upon, despite the frequency of this side effect [7]. Hematological toxicity is the most common adverse event and is responsible for fatal infections and bleeding episodes, especially during the first three months of treatment [8,9,10,11]. The incidence of grade ≥ 3 thrombocytopenia, neutropenia, and anemia subsequent to CAR-T cell treatment has been reported in 30–60% of patients [12]. Although some patients experience cytopenias resolving within a few days, others have many prolonged ones, harboring a high risk for severe or even fatal complications [10,12,13]. Pathophysiological mechanisms behind ICATH are not fully understood. Treatment of ICATH includes transfusion-supportive treatment, specific hematopoietic growth factor therapy, and stem cell boosts [8,14].

Eltrombopag is a synthetic thrombopoietin receptor agonist (TPO-RA) able to stimulate megakaryopoiesis [15]. Beyond its well-known efficacy to treat immune thrombocytopenia (ITP) [16], eltrombopag has been suggested to exert other immunomodulatory actions on bone marrow and thus contribute to restoring hematopoiesis in patients with severe aplastic anemia [17,18,19]. Eltrombopag has been able to restore platelet counts in disorders other than ITP [20]. TPO-RA has been used to reverse thrombocytopenia subsequent to CAR-T cell infusion in a few short case series. The results available so far are encouraging and suggest that the efficacy/safety profile of these agents is favorable. Nevertheless, the real-world experience is rather limited, and it is based predominantly on single or few center experiences and case reports [21,22]. In particular, there is no reliable data regarding the usefulness of eltrombopag in saving transfusion resources in CAR-T-treated patients with cytopenias. We tested the hypothesis that eltrombopag exhibits a favorable efficacy and safety profile when used to manage thrombocytopenia and, secondarily, other cytopenias in patients who had undergone CAR-T cell treatment. The aim of this work was to share the experience of the Spanish Group of Hematopoietic Transplant (GETH-TC) in this regard.

## 2. Materials and Methods

### 2.1. Patients and Study Design

A multicenter, observational, retrospective analysis was performed in Spanish hospitals by researchers belonging to the Spanish Group for Hematopoietic Transplantation and Cellular Therapy (GETH-TC). Patients to be recruited were those with a confirmed diagnosis of ALL or NHL, who underwent CAR-T cell therapy in the period of time between May 2019 and April 2024. Inclusion criteria were platelet transfusion dependence at day +30 or beyond after CAR-T therapy (platelet count < 20 × 10^9^/L, or higher in case of interventional procedure or special bleeding risk factors like coagulopathy, anticoagulant or antiplatelet treatment) [23]. Other inclusion criteria were the availability of complete clinical data, follow-up periods of at least 60 days after CAR-T cell infusion and 30 days after the administration of the first dose of eltrombopag, and written informed consent. Exclusion criteria were disorders involving dependence on immunosuppressant drugs or hormone therapy and severe and uncontrollable infection at study entry.

Treatment with eltrombopag (Revolade^®^, Novartis, Basel, Switzerland) was started from day 30 on after CAR-T infusion at a dose of 50 mg a day. Eltrombopag dose was increased every 2 weeks to 25 mg a day if the platelet count was lower than 20 × 10^9^/L and the patient was still dependent on platelet transfusion. The maximum dose of eltrombopag allowed to be used was 150 mg a day. In patients with platelet counts higher than 50 × 10^9^/L, eltrombopag was tapered in 25 mg a day every 2 weeks until stop treatment.

**Figure 1:**
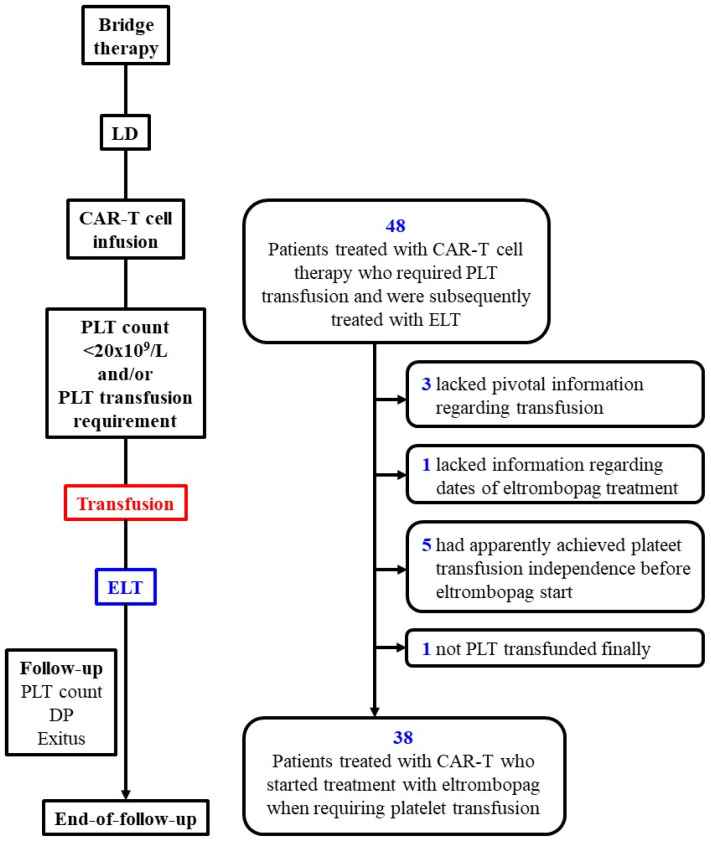
Flowchart diagram of the study and patients finally enrolled. DP, disease progression; ELT, eltrombopag; LD, lymphodepletion; mo, months; PLT, platelets.

Figure 1 shows the consort diagram of the study. Patients were followed from apheresis, including bridge therapy and lymphodepletion until the date of the end of follow-up, regardless of whether transfusion independence was achieved.

The study was approved by the ethics committee (Comité de Ético de la Investigación con medicamentos provincial de Sevilla) and was conducted in accordance with the 2013 revision of the Declaration of Helsinki. Ethical approval code number 0540-N-22.

### 2.2. CAR-T Cell Products

All patients were treated with autologous anti-CD19 CAR T-cell products: axicabtagene ciloleucel (axi-cel), tisagenlecleucel (tisa-cel), and ARI-0001. These are autologous anti-CD19 T-cell products containing a second-generation CAR. Axi-cel is developed using retroviral vectors and contains a CD28 co-stimulatory domain [24], while tisa-cel and ARI-0001 CAR19 constructs are created using lentiviral vectors and contain a CD137 (4-1BB) co-stimulatory domain [25,26].

### 2.3. Determinations and Analyses

Dates of onset and resolution of CRS and ICANS were reported. CRS and ICANS were graded according to the American Society for Transplantation and Cellular Therapy consensus guidelines [6]. Cytopenias subsequent to CAR-T treatment other than thrombocytopenia were also reported. Granulocyte colony stimulating factor (G-CSF) and erythropoietin use, platelet unit and red blood cell (RBC) transfusion requirement, bleeding episodes, dates of start and end of eltrombopag therapy and dose, reversal of thrombocytopenia and other cytopenias, toxicities during eltrombopag treatment, disease progression (DP) according to standardized criteria [27,28], and exitus, were reported.

The number of platelet units transfused according to days between the start of eltrombopag and achievement of sustained platelet count > 20 × 10^9^/L was assessed. Patients were also stratified into early or late responders to eltrombopag according to whether they reached platelet counts > 20 × 10^9^/L within the first 30 days of treatment or later, and transfusion requirement was compared between both groups. This limit of 30 days was chosen because it is the median time described to respond in previous publications [13,21,22].

### 2.4. Statistical Analysis

Discrete variables were summarized as numbers and percentages. Continuous variables were described by median (interquartile range [IQR]). The Mann-Whitney U-test was used to compare non-parametrically distributed continuous variables. Spearman’s rho coefficient was calculated to analyze the correlation between non-parametrically distributed variables.

## 3. Results

Thirty-eight patients were finally recruited for the study among 48 initially selected ones because they had been submitted to CAR-T cell therapy, developed transfusion-dependent thrombocytopenia, and were treated with eltrombopag (Figure 1). The median age was 59 years old, the number of men and women was similar, and almost 90% of patients had been diagnosed with DLBCL. Regarding CAR-T cell type, axi-cel was the most commonly used construct (Table 1). On the day of CART infusion, 18.5% of patients presented severe thrombocytopenia (<20 × 10^9^/L)

### 3.1. Recovery from Thrombocytopenia after Initiating Treatment with Eltrombopag

**Figure 2:**
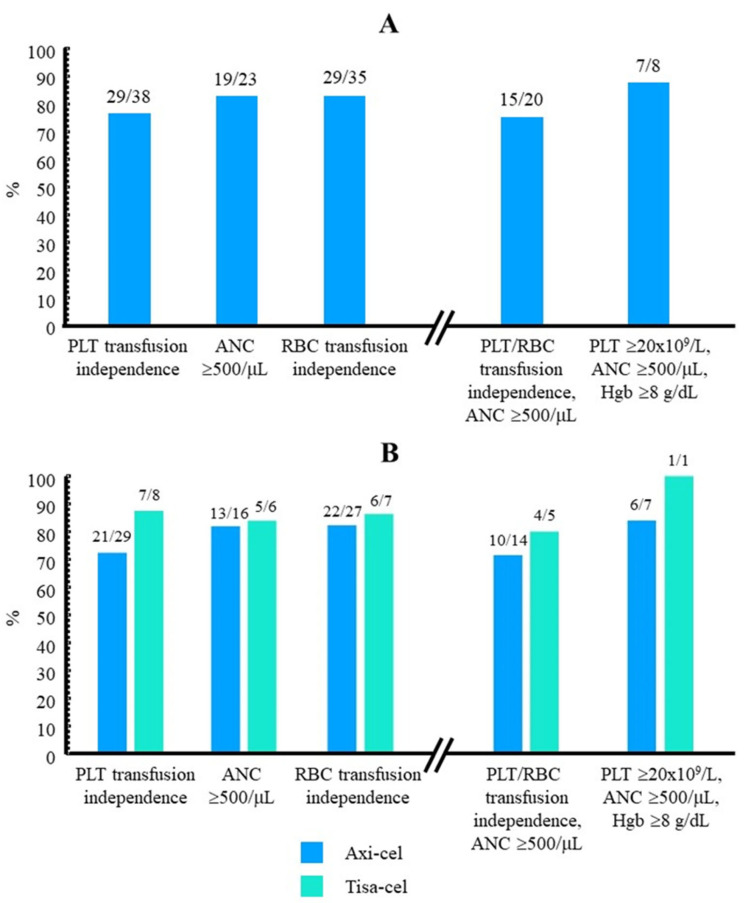
Cytopenia recovery subsequent to treatment with eltrombopag (**A**) The number of patients who recovered PLT/RBC transfusion independence or recovered from ANC < 0.5 × 10^6^/L, together with the total number of patients who required PLT/RBC transfusion or presented with ANC < 0.5 × 10^6^/L before the start of eltrombopag are indicated above each histogram (left). The number of patients who achieved simultaneously either platelet/RBC transfusion independence and ANC ≥ 0.5 × 10^6^/L or recovery from the three severe cytopenias upon eltrombopag treatment is also indicated (right). (**B**) The same calculations are presented after stratifying patients according to the type of CAR-T cell used. ANC, absolute neutrophil counts; axi-cel, axicabtagene ciloleucel; Hgb, hemoglobin; PLT, platelets; RBC, red blood cells; tisa-cel, tisagenlecleucel.

**Figure 3:**
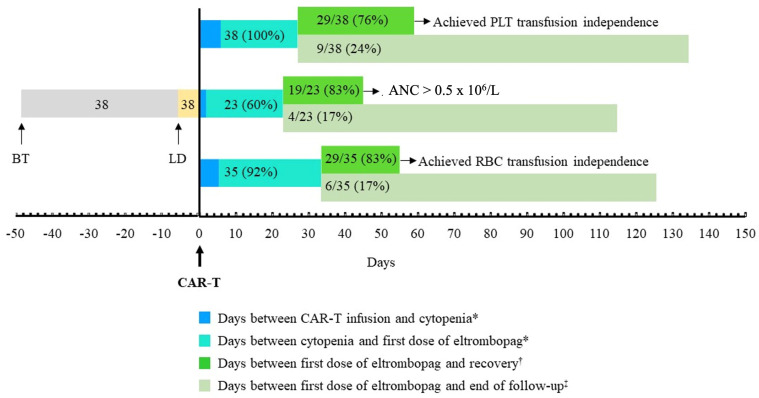
**Median time elapsed in each one of the phases of the study.** The median number of days was considered for each leg. The number of patients submitted to BT and LD, the number (percent) of patients who developed PLT transfusion dependence (above), severe neutropenia (middle), or RBC transfusion dependence (below), and the number (percent) of patients who achieved or did not achieve recovery before the end of the study are indicated. * Only those patients who developed platelet transfusion dependence (above), severe neutropenia (ANC < 0.5 × 10^6^/L, middle), or RBC transfusion dependence (below) were considered. ^†^ Only those patients who achieved platelet transfusion independence (above), reached ANC levels ≥ 0.5 × 10^6^/L (middle), or achieved RBC transfusion independence (below) were considered. ^‡^ Only those patients who did not recover from platelet transfusion requirement (above), ANC drop to levels < 0.5 × 10^6^/L (middle) or RBC transfusion requirement (below) were considered. ANC, absolute neutrophil counts; BT, bridging chemotherapy; LD, lymphodepleting chemotherapy; PLT, platelets; RBC, red blood cells.

**Figure 4:**
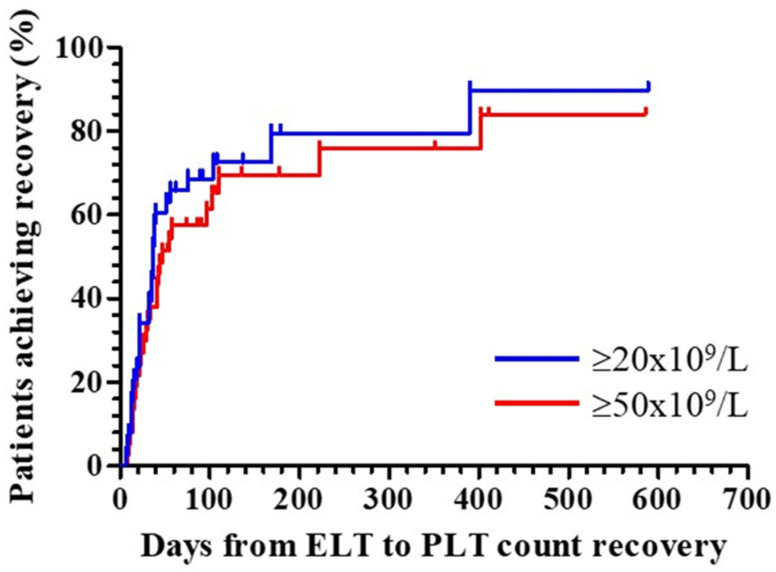
**Time course of platelet count recovery with eltrombopag.** Patients who presented with platelet counts < 20 × 10^9^/L after CAR-T cell therapy and started treatment with eltrombopag were considered. Kaplan-Meier curves were constructed considering the time elapsed between the first administration of eltrombopag and the recovery of platelet counts either ≥20 × 10^9^/L (blue line, *n* = 29) or ≥50 × 10^9^/L (red line, *n* = 26). Tick marks indicate those patients whose data were censored at the last follow-up date, either because of exitus or loss to follow-up, without having achieved PLT counts ≥ 20 × 10^9^/L (blue, *n* = 9) or >50 × 10^9^/L (red, *n =* 12). ELT, eltrombopag; PLT, platelets.

Table 2 summarizes the main results regarding eltrombopag use and the subsequent evolution of the platelet count. The median time from the first platelet transfusion after CART to the start of eltrombopag was 21 days (IQR: 7.5–55). This wide variation is related to protocol design; eltrombopag could be started from day +30 on at the discretion of the investigators. In several cases, investigators consider that there are other causes of thrombocytopenia, and this is an exclusion criterion of the study. In these cases, eltrombopag treatment was delayed. The median (IQR) time of follow-up after the first dose of eltrombopag administered was 122 (66, 398) days. By the end of follow-up, 29 out of 38 (76.3%) patients achieved a platelet count higher than 20 × 10^9^/L and recovered platelet transfusion independence (Table 2, Figure 2A and Figure 3). The time required to achieve this goal was 32 (IQR: 4, 38) days after the start of eltrombopag treatment. Figure 4 shows the time course of platelet count recovery once eltrombopag was started. Almost 80% of eltrombopag responders reached platelet counts higher than 20 × 10^9^/L in the first 40 days of treatment. The duration of eltrombopag treatment in the nine non-responder patients was 108 (63, 154) days (Table 2). Two of these patients died (1 infection, 1 ICANS), while on treatment, and eltrombopag was suspended at the time of disease progression in the other three cases.

Table 3 shows selected variables in the group of responders (*n* = 29) and non-responders (*n = 9*). Female sex and shorter time between the onset of transfusion-dependent thrombocytopenia and the start of eltrombopag seemed to be associated with a better response, although statistical significance was not reached. Finally, all-cause mortality among non-responders was notably higher than in those who got transfusion independence (77.8% and 44.8%, respectively, *p =* 0.130). Causes of death among non-responders included disease progression (3), infection (2), late ICANS (1), and a non-specified cause (1).

Regarding the efficacy of eltrombopag according to the CAR-T cell type used, only 1 out of 8 (12.5%) patients treated with tisa-cel did not achieve platelet transfusion independence, while this happened in 8 out of 29 (27.6%) who were administered axi-cel (Figure 2B). The number of days required to achieve platelet counts >20 × 10^9^/L was higher in axi-cel patients [32 (16, 47) days vs. 22 (8, 39) days treated with tisa-cel], although none of these comparisons were statistically significant.

The primary aim of eltrombopag therapy is to achieve and maintain platelet counts higher than 50 × 10^9^/L. Twenty-six out of thirty-eight (68.4%) patients had reached this goal by the end of follow-up (Table 2). Sixty-five percent reached counts higher than 50 × 10^9^/L in the first 45 days of eltrombopag (Figure 4). Median dose of eltrombopag was 100 mg a day (IQR: 50–150 mg a day). The whole of no responders received 150 mg a day of eltrombopag, and only 32% of responders were treated with the maximum dose.

### 3.2. Platelet Transfusion Requirement

**Figure 5:**
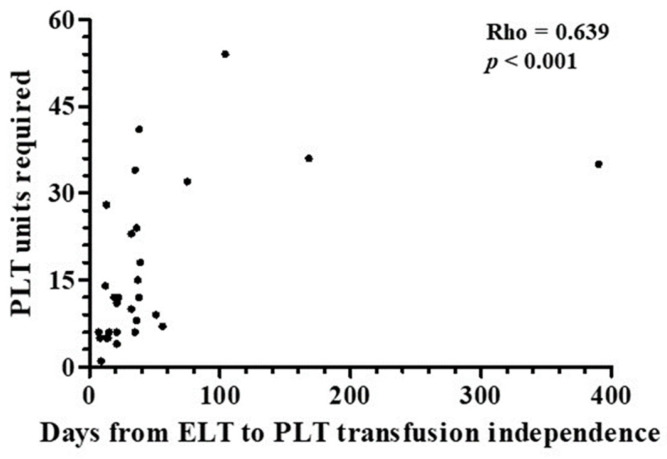
Correlation between total transfused platelet units and time between the start of eltrombopag and platelet transfusion independence achievement. The correlation between the total amount of transfused platelet units and the time elapsed between the start of eltrombopag therapy and the achievement of platelet transfusion independence is shown (*n* = 29). The one-tailed Rho Spearman test was used. ELT, eltrombopag; PLT, platelets.

**Figure 6:**
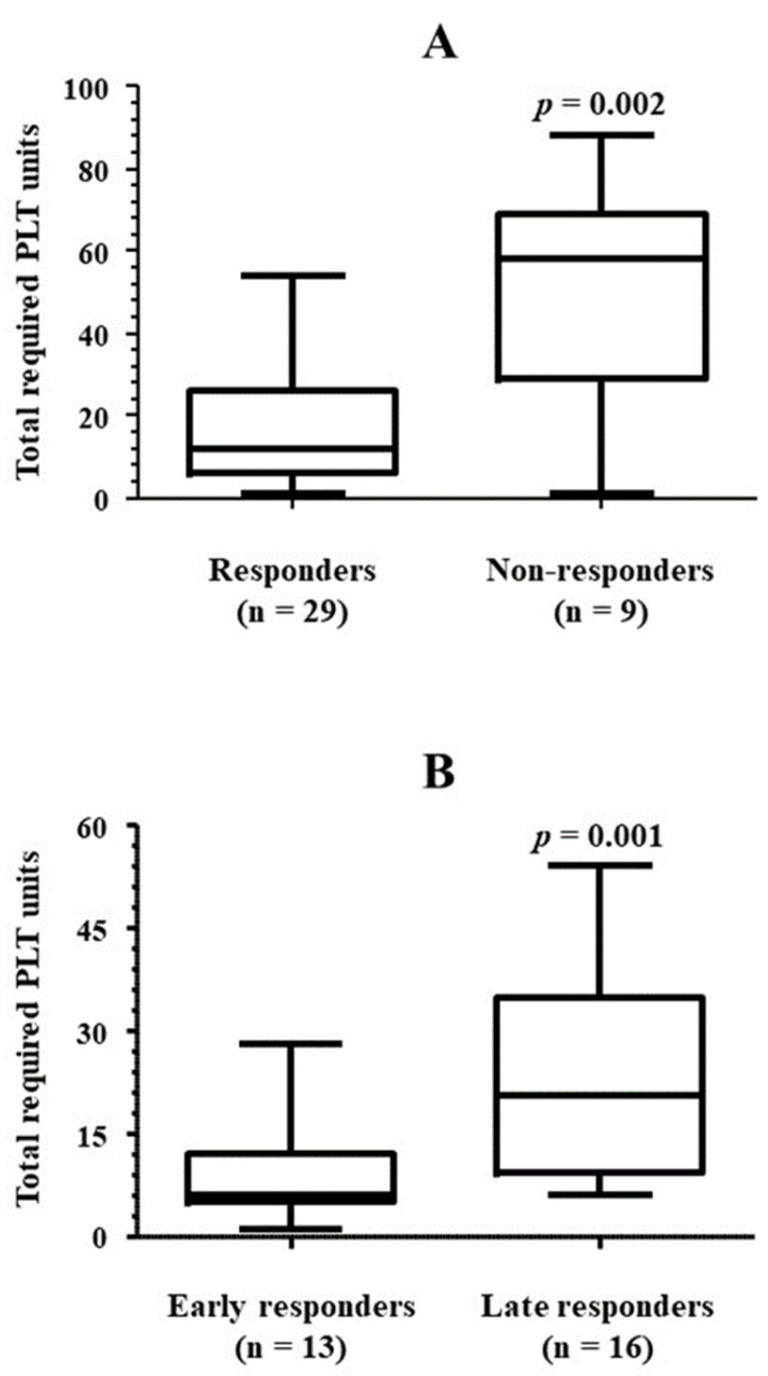
PLT transfusion requirement according to response to eltrombopag. (**A**) The amount of platelet units used in the patients who achieved platelet transfusion independence while in treatment with eltrombopag (responders) was compared against the platelet units used in those who had not achieved transfusion independence by the end of follow-up (non-responders). (**B**) The same comparison was performed within the group of responders, between those who achieved transfusion independence earlier (early responders) or later (late responders) than 30 days after the first dose of eltrombopag was administered. The one-tailed Mann-Whitney U test was used for comparisons. PLT, platelets.

**Figure 7:**
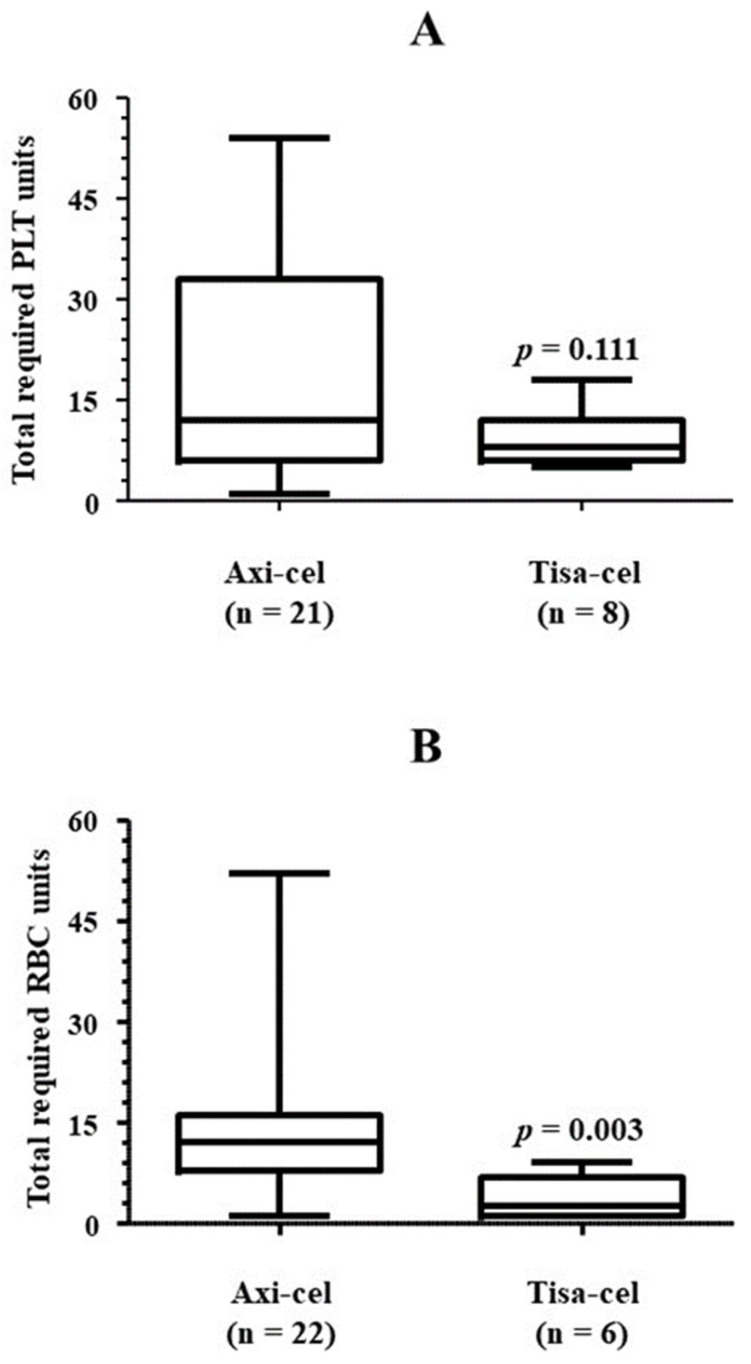
Transfusional requirement according to CAR-T cell type. The amount of platelet units (**A**) or RBC units (**B**) used in the patients who achieved platelet or RBC transfusion independence, respectively, is shown after categorizing them according to CAR-T cell type. The one-tailed Mann-Whitney U test was used for comparisons. Axi-cel, axicabtagene ciloleucel; PLT, platelets; RBC, red blood cells; tisa-cel, tisagenlecleucel.

Table 4 collects details regarding the number of platelet units used. All 29 patients who achieved platelet counts higher than 20 × 10^9^/L after starting treatment with eltrombopag recovered transfusion independence as well. The median number of platelet units transfused was 14, although requirements varied notably. There was a direct correlation between the total platelet units required and time from the administration of the first dose of eltrombopag to the achievement of transfusion independence (Rho = 0.639, *p* < 0.001) (Figure 5). Accordingly, the number of platelet units used by non-responders to eltrombopag treatment, i.e., those patients who did not achieve platelet counts higher than 20 × 10^9^/L by the end of the study, was significantly higher than that required by those patients whose platelet counts reached or exceeded the mentioned cut-off value before the last follow-up control (Figure 6A). Among responders, the platelet unit requirement was higher in those who required more than 30 days of eltrombopag therapy to achieve transfusion independence (Figure 6B). There was no correlation between platelet counts when the first dose of eltrombopag was administered and total platelet units used (Rho = 0.092, *p* = 0.315). Finally, those patients who were treated with axi-cel required more platelet units than those who used tisa-cel, although the difference did not reach statistical significance (Figure 7A). No patient received CD34 boost or allogenic transplantation of hematopoietic progenitors.

### 3.3. Bleeding Events during the Study

There was a total of three bleeding episodes, two of grade 3 from WHO and one of grade 4, that occurred when patients were under eltrombopag treatment (days 15, 70, and 16, respectively) (Table 5). None of these episodes were fatal. Patients suffered no other associated acquired coagulopathy. In these three patients, the time between detection of severe thrombocytopenia and the first dose of eltrombopag was 32 (18, 75) days, while this time was shorter, 15 (7, 53) days in those who had no bleeding episodes (*p* = 0.165). None of the three patients achieved transfusion independence before the end of the study, and they were considered non-responders.

### 3.4. Other Cytopenias and Concomitant Therapy

Twenty-three (62.2%) and 13 (39.4%) patients had severe neutropenia [absolute neutrophil count (ANC) < 0.5 × 10^6^/L] and severe anemia (hemoglobin < 80 g/L), respectively, when treatment with eltrombopag was started. At this time, 35 out of 38 (92.1%) patients required RBC transfusion, and 62.2% presented neutropenia grade 4 (Table 6). Half of the patients in the cohort were also treated with erythropoietin, although this treatment was not used in eight patients who had severe anemia. Nineteen out of twenty-three (82.6%) and 29 out of 35 (82.9%) patients recovered from neutropenia and RBC transfusion independence by the end of follow-up, needing 22 (10, 31) days and 21 (0, 43) days, respectively (Table 6, Figure 2A and Figure 3). All 19 patients who recovered ANC higher than 0.5 × 10^6^/L also achieved ANC values of 1 × 10^6^/L after a median period of time of 6 days. With regard to hemoglobin, 11 out of 13 (84.6%) patients who had baseline levels < 80 g/L had recovered this threshold by the end of follow-up (Table 6). A trend toward better cytopenia recoveries was again envisaged when tisa-cel was used instead of axi-cel (Figure 2B). Remarkably, the number of RBC units required by those patients was significantly higher when axi-cel was used (Figure 7B).

### 3.5. Recovery When Platelet/RBC Transfusion Dependence and Severe Neutropenia Presented Simultaneously

There were 20 patients who presented with platelet/RBC transfusion dependence as well as ANC < 0.5 × 10^6^ when they were administered the first dose of eltrombopag. Fifteen of them (75.0%) had achieved platelet/RBC transfusion independence and reached ANC higher than 0.5 × 10^6^ before the end of follow-up (Figure 2A). The percentage of whole recovery was 80% in tisa-cel-treated patients and 71.4% in axi-cel-treated patients (Figure 2B). Eight patients presented with signs of aplasia, with simultaneous platelet counts < 20 × 10^9^/L, ANC < 0.5 ×10^6^ and hemoglobin < 80 g/L. Upon treatment with eltrombopag, seven (87.5%) of them recovered from severe thrombocytopenia, neutropenia, and anemia by the last follow-up date (Figure 2A).

### 3.6. Safety

#### 3.6.1. Adverse Events Associated with CAR-T Cell Therapy

CRS and ICANS were developed by 32 (84.2%) and 21 (55.3%) patients, respectively, generally within the first week after CAR-T cell infusion (Table 7). The median time from onset to resolution was lower than 7 days in both cases, although one patient was reported to die because of late ICANS. Up to 50% of patients in the cohort had at least one episode of infection during the study, and six of them died (sepsis 2, varicela-zoster virus 1, COVID-19 pneumonia 3; Table 7).

#### 3.6.2. Adverse Events Associated with Eltrombopag

No thromboembolic events were reported in the period between the start of eltrombopag treatment and the last follow-up date. Only two patients had toxicities when in treatment with eltrombopag, namely cholestasis/transaminitis and hyperbilirubinemia (Table 7). Both cases were of mild-to-moderate severity and transient. In the first case, eltrombopag (50 mg/day) was suspended and resumed at the same dosage after resolution. In the second one, the dose was decreased to 100 mg/day until resolution and resumed at 150 mg/day without any complication.

### 3.7. Outcomes

After a median follow-up of 175 (99, 489) days, 12 out of 38 (31.6%) patients had DP. This occurred at a median time of 98 (89, 131) days since CAR-T cell therapy was administered. Twenty (52.6%) patients died by the end of the study, mainly because of DP (60%) and infection (30%; Table 8).

## 4. Discussion

Prolonged thrombocytopenia subsequent to CAR-T cell treatment has been reported in clinical and real-world studies, with platelet counts < 50 × 10^9^/L or <10 × 10^9^/L in 24–26% and 5–7% patients at post-infusion days 30 and 90, respectively (reviewed in [21]). Thrombocytopenia and other cytopenias have been described to follow a biphasic temporal course, with a nadir of platelets and neutrophils at approximately 6 weeks [8]. Although spontaneous recovery after a few weeks or months has been described [21], CAR-T-treated patients may be at an unacceptably high risk of bleeding and infection until this occurs. TPO-RAs, like eltrombopag, romiplostim, and, more recently, avatrombopag, are indicated to restore platelet counts in ITP and severe aplastic anemia [16] and have also been successfully used after allogeneic stem cell transplantation or chemotherapy [29,30].

In our cohort, more than 75% of patients recovered from platelet transfusion requirement and achieved platelet counts higher than 20 × 10^9^/L after a median time of 32 days on eltrombopag. Considering that the median time that elapsed between the first platelet transfusion and the first dose of eltrombopag was 21 days, patients who responded to eltrombopag would have been at bleeding risk for a period of time in the median range of 50–60 days. Since the persistence of thrombocytopenia for periods of 90 days or more in CAR-T cell-treated patients is not infrequent [13], our findings suggest that eltrombopag may be useful to shorten the bleeding risk period. Furthermore, 26 out of the 29 patients who recovered from platelet transfusion requirement also achieved platelet counts > 50 × 10^9^/L, i.e., the target value pursued by this treatment in other indications [31].

We reported 3 (7.9%) patients who had a bleeding episode while on treatment with eltrombopag, which lasted for a median of 68 days. None of these was fatal. Another series of CAR-T cell-treated patients who did not use TPO-RAs reported bleeding incidences ranging between 9 and 42% [32,33], with bleeding found to be associated with reduced survival [32].

The literature addressing the use of TPO-RAs in the CAR T-cell setting is limited (reviewed in [7]). To date, only one retrospective real-world study with a sample size in our range has been reported. This cohort of 42 patients treated with CAR-T cells also received eltrombopag because of cytopeniaa. The time between CAR-T cell administration and the first dose of eltrombopag, as well as treatment duration were similar to ours. After 60 days on eltrombopag, the proportion of patients with grades 3–4 thrombocytopenia dropped from 93% to 46%. The bleeding event incidence did not differ from that reported in CAR-T cell-infused patients not treated with eltrombopag [22]. Two studies enrolling a total of 17 TPO-RA-treated patients, 10, 4, and 1 of whom received, respectively, eltrombopag, romiplostim, or both, also showed results similar to ours with regard to time to platelet transfusion independence [21,34]. Finally, one case report described platelet transfusion independence immediately after the start of romiplostim [35].

Studies addressing the suitability of the use of eltrombopag in CAR-T cell-treated patients do not provide clues regarding hallmarks to draw distinctive profiles of responders and non-responders. In our cohort, non-responders were on eltrombopag for a median number of days largely higher than that required by responders to achieve platelet counts > 20 × 10^9^/L. This finding invites us to think that the failure of treatment was not due to an early interruption. On the other hand, despite the majority of responses being early responses, close to 30% of responders need a longer period of treatment. This fact should be taken in mind before considering eltrombopag treatment a failure. Finally, it is important to exclude other causes of thrombocytopenia if eltrombopag is not useful. Three of the non-responder patients stopped eltrombopag when DP was documented, and another two patients died while still on treatment, suggesting that no response might be associated with a serious condition.

On the other hand, although the sample sizes, especially those of non-responders, do not allow us to draw reliable conclusions, female patients seemed to respond better, and the time between the onset of thrombocytopenia and the start of therapy might have also influenced the response. Patients treated with tisa-cel seemed to respond better than those infused axi-cel constructs. Axi-cel products, although very efficient, have been described to be more toxic and exert more proinflammatory actions than those based on tisa-cel, thus challenging cytopenia recovery in DLBCL and ALL patients [36,37,38].

Platelet transfusion is an essential part of supportive care subsequent to CAR-T cell therapy, which is required by more than 65% of patients after CAR T-cell infusion and may become a concern regarding resource consumption when thrombocytopenia persists in time [9,39]. We found that the number of platelet units required was closely associated with the time from the start of eltrombopag and the achievement of transfusion independence. The highest expense corresponded to non-responders. Thus, on the one hand, the use of eltrombopag may contribute to saving health resources. On the other hand, profiling responders and non-responders may help to identify candidates for eltrombopag therapy and should be a task for future studies. By now, we have no information to identify responders, and there are no responders, and there are no early or late responders to TPO-RA. Finally, in accordance with our findings, axi-cel users consumed more platelet units than those treated with tisa-cel products.

While on treatment with eltrombopag, more than 80% of patients with severe neutropenia recovered ANC counts > 0.5 × 10^6^/L in a median time of 22 days. In the following week of treatment, all of them presented with ANC counts > 1 × 10^6^/L. Wesson et al. reported post-CAR-T cell therapy leukopenia of grades 3–4 in 74% of patients when eltrombopag was started and documented a drop to 15% after approximately 60 days of treatment [22]. ANC was also reported to improve with eltrombopag in small-sized studies [21,34,35]. A small subset of patients has been described to present with persistent, severe neutropenia [40], and pivotal trials have reported an infection incidence of 19–69%, of which 5–32% were severe [41]. Thus, eltrombopag, used along with G-CSF, may be useful to prevent subsequent risk of severe infection. Nevertheless, somewhat surprisingly, eltrombopag has been associated with an increase in infection rate in CAR-T cell-treated patients [22], and we reported infection episodes in up to 50% of our patients, 26% of which were fatal. The potential association between eltrombopag and infection deserves further investigation, but probably patients treated with TPO-RA are those with more severe and longer cytopenias, being at higher risk of infections because of these circumstances, not because of TPO-RA treatment.

Thirty-five out of 38 patients required RBC transfusion after CAR-T cell infusion. More than 80% of them achieved RBC transfusion independence after a median period of 29 days with eltrombopag. Wesson et al. reported a less noteworthy improvement, with the proportion of patients with anemia of grade 3–4 decreasing from 41% to 31% after around 60 days on eltrombopag [22]. Other available studies have also found beneficial effects of this treatment on RBC counts [21,34,35]. We also found that the proportion of axi-cel-treated patients who recovered RBC transfusion independence was lower than that observed among those infused tisa-cel products. Remarkably, the total amount of RBC units required by axi-cel users was more than 4-fold the amount transfused to tisa-cel users, also when the analysis was limited to those who did not require RBC transfusion at the last follow-up date. Once more, this finding is in accordance with previous reports acknowledging a higher toxicity burden associated with axi-cel use [36,37,38].

Aplastic phenotype has been described as one of the possible patterns of hematopoietic reconstitution after CAR T-cell therapy [8]. Eltrombopag has been successfully used to manage severe aplastic anemia, preferably at a dose of 150 mg/day [19,42]. In our cohort, eight patients presented with this phenotype, usually within the first 15 days after CAR-T cell infusion. All were treated with a dose of 150 mg/day, and seven of them recovered simultaneously from all cytopenias. These findings are in accordance with those described by Beyar-Katz et al., who reported simultaneous recovery from all severe cytopenias in 5 out of 6 CAR-T cell-infused patients who were treated with eltrombopag at 150 mg/day [34]. TPO-RAs have been found to improve trilineage hematopoiesis in aplastic anemia patients despite the presence of high levels of endogenous thrombopoietin [43], which suggests that analogs may exert this effect using alternative anti-inflammatory mechanisms [44].

NHL patients are at increased risk of venous thromboembolism, especially those with DLBCL [45]. VTE is also a well-known complication in ALL patients [46]. On the other hand, although not commonly, thrombotic events have been described in patients after CAR-T cell treatment, and close monitoring is recommended [47,48]. TPO-RAs have been associated with higher thrombosis risk in ITP patients, even in those with low platelet counts [49,50]. With this background, the occurrence of thrombotic events in CAR-T-infused patients treated subsequently with eltrombopag should be carefully assessed. Neither venous nor arterial ischemic episodes were reported in our 38 patients while on eltrombopag therapy. Events were not reported either in the 42 patients of the other large real-world series [22].

Only two mild-to-moderate toxicities were reported in our patients while on eltrombopag, both associated with hepatobiliary damage, as previously described by others [22], and of a transient nature. Therefore, eltrombopag shows a favorable safety profile to be used to recover from thrombocytopenia and other cytopenias developed after CAR-T cell infusion.

Our study has limitations, many of them inherent to the retrospective nature of the study. Some degree of heterogeneity among participating centers in terms of disease management should not be ruled out. Comparison against CAR-T cell treated patients who were not treated with eltrombopag despite presenting with severe thrombocytopenia was not possible: there is a bias in that physicians tend to limit eltrombopag use to those patients with a more severe clinical condition and, thus, the extent of the effect of eltrombopag regarding recovery from cytopenias and transfusion requirement cannot be precisely determined.

The small number of non-responders to eltrombopag, in terms of recovery from severe thrombocytopenia, precluded us from drawing reliable profiles of responders and non-responders. Among these, exitus may have prevented the achievement of platelet count recovery, although the time that elapsed after the first dose of eltrombopag was administered was largely longer than the time required by eltrombopag responders to achieve platelet transfusion independence. Finally, although the results obtained with tisa-cel in terms of cytopenia recovery and transfusion requirement seemed to be better than those of axi-cel, the small number of patients who were treated with the former made it hard for us to establish more reliable conclusions.

## 5. Conclusions

In summary, we have assessed the efficacy/safety profile of eltrombopag in managing CAR-T cell-treated patients who developed thrombocytopenia and other cytopenias in a real-world cohort of patients. Our results suggest that eltrombopag could be a valid tool for this scenario. Although the time required to recover from severe thrombocytopenia in eltrombopag-treated patients is much shorter than those described elsewhere for spontaneous recovery, at least in a subset of CAR-T cell-treated patients, the retrospective nature of the study prevents us from formally stating that eltrombopag contributes to shortening the duration of platelet recovery and platelet transfusion requirement. Indeed, our findings firmly encourage the design of prospective studies to confirm this benefit and, secondarily, to undertake other studies associated with other cytopenias.

## Figures and Tables

**Table 1:** Baseline features of patients under CAR-T treatment treated with eltrombopag.

Variable	Value
Age, median (IQR)	59.0 (50.1, 64.3)
Sex (female)	17/38 (44.7)
Disorder	
DLBCL	34/38 (89.5)
ALL	3/38 (7.9)
FL	1/38 (2.6)
DLBCL Characteristics Stage disease 3 or 4 Extranodal disease Bulky disease IPI 3–5 CNS disease 3 lines of treatment or more	28/34 (83%) 21/34 (61.8%) 11/34 (33.8%) 18/34 (52.9%) 2/34 (5.9%) 19/34 (55.9%)
Disease Stage Before CART Complete Remission Partial remission Stable Disease Progression Disease	2/38 (5.4%) 4/38 (10%) 12/38 (31.9%) 20/38 (52.7%)
Bridge therapy:	30/38 (78.9)
Type	
R-GEMOX	12/30 (40.0)
R-GDP	6/30 (20.0)
BR	3 */30 (10.0)
R-ICE	1/30 (3.3)
Polatuzumab and BR	1/30 (3.3)
R-ESHAP	1/30 (3.3)
Inotuzumab	1/30 (3.3)
R-MINE	1/30 (3.3)
Radiotherapy	1/30 (3.3)
R-MTX	1/30 (3.3)
Burkimab	1/30 (3.3)
Several strategies	1/30 (3.3)
FluCy lymphodepletion standard ”	38/38 (100)
CAR-T type	
AXI-CEL	29/38 (76.3)
TISA-CEL	8/38 (21.0)
Other	1/38 (2.6)

Results are *n/N* (%), except otherwise indicated. * Combined with CFX or DEXA, one case each. ALL; acute lymphoblastic leukemia; AXI-CEL, axicabtagene ciloleucel; BR, bendamustine and rituximab; CFX, cyclophosphamide; DEXA, dexamethasone; DLBCL, diffuse large B cell lymphoma; FL, follicular lymphoma; FluCy, fludarabine, and cyclophosphamide; IQR, interquartile range; R-ESHAP, rituximab combined with etoposide, methylprednisolone, cytarabine, and cisplatin; R-GDP, rituximab combined with gemcitabine, dexamethasone, and cisplatin; R-GEMOX, rituximab combined with gemcitabine and oxaliplatin; R-ICE, rituximab combined with ifosfamide, carboplatin and etoposide; R-MINE, rituximab combined with ifosfamide, mitoxantrone, etoposide and prednisone; R-MTX, rituximab combined with methotrexate; TISA-CEL, tisagenlecleucel. “FluCy lymphodepletion standard: Cyclophosphamide 500 mg/m^2^/d IV 2 days (days 3 and 2) and fludarabine 25 mg/m^2^/day IV 3 days (days 4 to 2).

**Table 2:** Evolution of thrombocytopenia subsequent to treatment with eltrombopag.

Variable	Value
Time from CAR-T infusion to PLT transfusion requirement, days	6.0 (2.0, 21.5)
Platelet count when the first dose of eltrombopag was administered, ×10^9^/L	12.5 (9.0, 18.1)
Time from first platelet transfusion to start of eltrombopag, days	21.0 (7.5, 55.0)
Eltrombopag dose, mg/d	
Initial	50 (50, 50)
Maximum	150 (50, 150)
Time on eltrombopag treatment, days	68 (48, 154)
Follow-up since the start of eltrombopag, days	122 (66, 398)
Patients who recovered counts at follow-up end and time required	
Achieved platelet count ≥ 20 × 10^9^/L, n/N (%)	29/38 (76.3)
Time from start of eltrombopag to platelet count ≥ 20 × 10^9^/L, days	32 (14, 38)
Achieved platelet count ≥ 50 × 10^9^/L, n/N (%)	26/38 (68.4)
Time from start of eltrombopag to platelet count ≥ 50 × 10^9^/L, days	33 (19, 57)
Patients who did not reach counts ≥ 20 × 10^9^/L at follow-up end	
Time on treatment with eltrombopag, days	108 (63, 154)
Follow-up since the start of eltrombopag, days	108 (82, 158)

Results are median (IQR), except otherwise indicated. IQR, interquartile range.

**Table 3:** Distribution of selected variables in patients stratified according to response to eltrombopag.

Variable	Responders (n = 29)	Non-Responders (n = 9)	*p*
Age	59.8 (48.0, 64.5)	58.4 (53.8, 67.3)	0.810
Sex (female), n/N (%)	15/29 (51.7)	2/9 (22.2)	0.148
Diagnosis (DLBCL), n/N (%)	27/29 (93.1)	7/9 (77.8)	0.233
Cytopenia before CART infusion (%)	8/29 (27.5)	2/9 (22)	0,573
Disease in progression before CART (%)	15/29 (51.7)	5/9 (55.5)	0,945
Bridge therapy (%)	6/29 (20.7)	2/9 (22)	0,338
PLT count at ELT start (×10^9^/L)	12 (9, 15)	15 (9, 23)	0.633
Time between PLT transfusion start and ELT start, days	15 (6, 46)	36 (22, 68)	0.196
DP before the end of the study, n/N (%)	9/29 (31.0)	3/9 (33.3)	1.000
Exitus before the end of the study, n/N (%)	13/29 (44.8)	7/9 (77.8)	0.130

Results are median (IQR) except otherwise specified. Responders and non-responders were patients who either achieved or did not achieve platelet transfusion independence by the last follow-up visit. Comparisons were performed using the two-tailed Mann-Whitney U test and the two-tailed Fisher exact test for continuous and categorical variables, respectively. DP, disease progression; ELT, eltrombopag; IQR, interquartile range; PLT, platelets.

**Table 4:** Platelet transfusion requirement.

Variable	Value
Patients requiring PLT transfusion at the start of eltrombopag *, *n/N* (%)	38/38 (100)
Time from platelet count < 20 × 10^9^/L to start of eltrombopag, days	21 (7, 55)
Time between first and last PLT transfusion, days	55 (28, 129)
Total transfused PLT units	14 (6, 34)
Patients who achieved PLT transfusion independence at follow-up end, *n/N* (%)	29/38 (76.3)

Results are median (IQR), except otherwise indicated. * Transfusion was considered for a platelet count threshold < 20 × 10^9^/L, or larger in the event of suspicion of acute platelet drop or bleeding risk, according to AABB recommendations [23]. IQR, interquartile range; PLT, platelets.

**Table 5:** Bleeding events WHO grade 2 or more reported during the study.

Bleeding While on Eltrombopag Treatment	Value
Patients, *n/N* (%)	3/38 * (7.9)
WHO grade	3.0 (3.0, 4.0)
Fatal, *n/N* (%)	0/3 (0)
Location, *n/N* (%)	
Several ^†^	1/3 (33.3)
Subdural	1/3 (33.3)
Nasopharynx	1/3 (33.3)
Features of patients who had a bleeding episode	
Platelet count before the start of eltrombopag	18.5 (20.0, 27.0)
Time from platelet count < 20 × 10^9^/L to start of eltrombopag, days	32 (18, 75)
Time on eltrombopag treatment when bleeding occurred, days	16 (15, 70)

Results are median (IQR), except otherwise indicated. * Another patient had a non-fatal grade 4 brain hemorrhage 5.5 weeks after platelet count < 20 × 10^9^/L was detected and 2.5 weeks before eltrombopag was started. ^†^ Mucosal and digestive bleeding and petechiae. IQR, interquartile range; PLT, platelets; TCP, thrombocytopenia; WHO, World Health Organization.

**Table 6:** Evolution of other cytopenias subsequent to treatment with eltrombopag.

Variable	Value
Severe neutropenia * at start of eltrombopag, *n/N* (%)	23/37 ^†^ (62.2)
ANC at the start of eltrombopag, cells × 10^6^/L	1 (0, 8.4)
Use of G-CSF, *n/N* (%)	37/37 ^†^ (100)
Time on treatment, months	1.50 (1.00, 2.75)
Patients who recovered ANC by follow-up end and time required	
From <0.5 × 10^6^/L to ≥0.5 × 10^6^/L, *n/N* (%)	19/23 (82.86)
Time from start of eltrombopag to ANC ≥ 0.5 × 10^6^/L, days	22 (11, 31)
From <0.5 × 10^6^/L to ≥1 × 10^6^/L, *n/N* (%)	19/23 (82.86)
Time from start of eltrombopag to ANC ≥ 1 × 10^6^/L, days	28 (18, 35)
Severe anemia ^‡^ at start of eltrombopag, *n/N* (%)	13/33 ^§^ (39.4)
Hemoglobin at start of eltrombopag, g/L	82 (76, 93)
Use of EPO	19/38 (50.0)
Time on treatment, months	2.00 (2.00, 3.00)
Patients requiring RBC transfusion, *n/N* (%)	35/38 (92.1)
Total transfused RBC units	
Overall	12.0 (4.0, 27.0)
In patients with hemoglobin < 80 g/L	12.0 (6.5, 17.5)
Patients who achieved RBC transfusion independence, *n/N* (%)	29/35 (82.9)
Time from start of eltrombopag to RBC transfusion independence, days	29 (17, 44)
Patients who achieved hemoglobin ≥ 80 g/L by follow-up end, *n/N* (%)	11/13 (84.6)
Patients with severe pancytopenia ^¶^ at start of eltrombopag, *n/N* (%)	8/38 (21.0)
Patients who recovered all lineages by follow-up end, *n/N* (%)	7/8 (87.5)

Results are median (IQR), except otherwise indicated. * As defined by ANC < 0.5 × 10^6^/L. In 5 cases, this condition was present when CAR-T cells were infused; in 15 and 5 cases, this condition presented early (within the first 30 days after CAR-T cell infusion) and late (later than the first 30 days), respectively. ^†^ Data missing from 1 patient. ^‡^ As defined by hemoglobin < 80 g/L. ^§^ Data missing from 5 patients. ^¶^ Platelet count < 20 × 10^9^/L, ANC < 0.5 × 10^6^/L and hemoglobin < 80 g/L. ANC, absolute neutrophil count; EPO, erythropoietin; G-CSF, granulocyte colony-stimulating factor; IQR, interquartile range; RBC, red blood cell.

**Table 7:** Adverse events other than cytopenias.

Adverse Events Before and/or during Eltrombopag Treatment	
CRS	
Patients, *n/N* (%)	32/38 (84.2)
Time from CAR-T cell infusion to onset, days	2 (1, 4)
Grade	2 (1, 2)
Time from onset to resolution, days	5 (4, 9)
ICANS	
Patients, *n/N* (%)	21/38 (55.3)
Time from CAR-T cell infusion to onset, days	6 (5, 8)
Grade	2 (2, 3)
Time from onset to resolution, days	4 (2, 7)
Infection, *n/N* (%)	19/38 (50.0)
Fatal, *n/N* (%)	6/19 (26.3)
Toxicities during eltrombopag treatment	
Patients with toxicities requiring eltrombopag suspension, n/N (%)	1/38 (2.6)
Type of toxicity	
Cholestasis, transaminitis *, *n/N* (%)	1/1 (100)
Patients with other toxicities not requiring hospitalization or suspension, *n/N* (%)	1/38 (2.6)
Type of toxicity	
Hyperbilirubinemia ^†^, *n/N* (%)	1/1 (100)

Results are median (IQR), except otherwise indicated. * One episode; eltrombopag was resumed when toxicity was resolved. ^†^ One episode; eltrombopag dose was lowered to 100 mg/day until resolution. CRS, cytokine release syndrome; ICANS, immune effector cell-associated neurotoxicity syndrome; IQR, interquartile range.

**Table 8:** Outcomes by follow-up end.

Variable	Value
Follow-up from CAR-T cell infusion to end of study, days, median (IQR)	175 (99, 489)
DP by follow-up end	12/38 (31.6)
Time from CAR-T infusion to DP, days, median (IQR)	98 (89, 131)
Exitus by follow-up end	20/38 (52.6)
Cause	
Disease progression	12/20 (60.0)
Infection	6/20 (30.0)
Late ICANS	1/20 (5.0)
Unspecified	1/20 (5.0)

Results are *n/N* (%), except otherwise indicated. DP, disease progression; IQR, interquartile range.

## Data Availability

Datasets are available upon request to the corresponding author.
